# Quantitative assessment of Ni^+^ and He^+^ ion irradiation damage in a tungsten heavy alloy under the simulated nuclear fusion environment

**DOI:** 10.1038/s41598-025-89532-w

**Published:** 2025-02-27

**Authors:** James V. Haag, Yucheng Fu, Weilin Jiang, Bethany E. Matthews, Matthew J. Olszta, Danny J. Edwards, Wahyu Setyawan

**Affiliations:** 1 Energy and Environmental Directorate, Pacific Northwest National Laboratory, Richland, WA USA; 2 Physical and Computational Sciences Directorate, Pacific Northwest National Laboratory, Richland, WA USA

**Keywords:** Metals and alloys, Characterization and analytical techniques

## Abstract

A 90W-7Ni-3Fe (wt.%) tungsten heavy alloy has been sequentially Ni^+^ and He^+^ ion irradiated at 700 °C to simulate the high temperature irradiation environment of a fusion reactor interior. W/Ni–Fe-W dual-phase alloys have been proposed to serve as plasma facing materials and require detailed investigation of their behavior under fusion relevant conditions to assess their overall applicability. To evaluate material performance under five years of simulated fusion reactor service, microstructural characterization of the nanoscale defect distribution has been performed on both constituent phases, revealing peak swelling in the W phase of approximately 0.03%. The γ-phase (Ni–Fe-W) is found to swell approximately 0.68% under the same irradiation conditions, indicating significant cavity formation and growth. Additionally, a novel multi-projection imaging approach has been applied to determine the extent of damage segregation along the dual-phase W-to-γ interface and exposes that these interfaces act as sink sites for the accumulation of cavities. Interphase boundaries are noted to possess an 11.8% areal coverage of defects along the boundary plane, primarily on the γ-phase side of the boundary. The accumulation of cavities at these interphase boundaries is anticipated to adversely affect overall material toughness, and this work reveals a pressing need for mechanical property testing of irradiated W–Ni-Fe dual-phase alloys.

## Introduction

As nuclear fusion begins to enter the engineering era, there is a pressing need for experimental data on the performance of materials under the fusion environment. With the current lack of long-term operational test reactors, the fusion materials community continues to push for the implementation of a fusion prototypic neutron source (FPNS). This is needed to produce the characteristic 14 MeV neutrons with sufficient flux to accurately observe the resultant damage structures in plasma facing materials (PFM) and other component materials, but until these facilities are brought online, the community must employ surrogate irradiations to simulate the fusion environment. The two primary schools of thought for simulating the fusion environment are a subject of considerable debate, but boil down to the use of ion irradiations to produce the displacement damage and gas production, or the use of a lower-energy spectrum of neutrons^1–3^. Rather than become entrenched in a debate over best-practices for specimen irradiation, it is clear that both techniques generate useful data to advance the current understanding of radiation tolerance, but both techniques also produce fundamentally different damage than that of an operational fusion reactor.

In an effort to provide the data necessary to support characterization efforts of fusion reactor materials, a material proposed as a PFM has been subjected to the simulated fusion environment by applying sequential self-ion (Ni^+^) and He^+^ ion irradiations at high temperature (700 °C). The material employed in this study is an as-sintered 90W-7Ni-3Fe (wt.%) tungsten heavy alloy (WHA) and has been the subject of ongoing investigation^4–11^ since receiving promising experimental results in the ASDEX Upgrade in 2016 experimental campaigns^12,13^. The dual-phase nature of this alloy takes advantage of the multiple benefits of pure W, the current leading candidate for the divertor material, with the addition of a Ni–Fe based ductile phase which serves to improve the poor fracture toughness of polycrystalline tungsten. While the overall ductility and fracture toughness of the WHA is significantly increased, introducing Ni and Fe is ultimately a trade-off in terms of material performance, adding potential neutron activity and void swelling concerns. Introduction of a ductile phase and its bearing upon mechanical properties and waste disposal rating have been covered in prior works^8,14–17^, but to gauge the applicability of this material and its structural response to the irradiation conditions of a fusion reactor, detailed characterization of WHA behavior under applied irradiation must necessarily be performed. Due to the extremely shallow penetration depth of ion irradiation, this analysis requires extreme site specificity and sensitivity to nanoscale defect structures. Prior analyses of this alloy under the same irradiation conditions revealed the development of W-based carbides at the bi-phase interface, likely resulting from contamination during the irradiation process^9^, but several outstanding questions remain in developing a mechanistic understanding the response of WHAs to applied irradiation and generating quantitative characterization data to represent this incurred damage.

Of particular interest in the assessment of WHA response to the simulated fusion environment is the production of a depth-resolved and quantitative investigation of material swelling, cavity morphology, and density; then performing this analysis in both bulk phases as well as at the bi-phase interface. Preliminary results indicate the development of cavities at the interphase boundaries (IPBs) between the W and ductile phases, which could contribute to material embrittlement^18,19^. This study has therefore been designed to employ transmission electron microscopy (TEM) to determine the effects of applied irradiation conditions mimicking the fusion environment on the overall manifestation of defect structures in the WHA system. As quantitative characterization is required to accurately portray the nature of material response, a concerted effort has been made to represent the nature of the incurred damage numerically. In particular, a novel approach has been applied to deconvolute projection effects in the characterization of interfaces under irradiation, providing a quantifiable sense of damage segregation at the IPBs, in which the strong cohesion of the IPBs is key to the excellent toughening, and thereby mechanical properties in the WHA system^8,10^. This is done to assess the nature of material performance under high temperature irradiation in the WHA system as well as to provide insight into its expected performance as a plasma facing material.

## Results

**Fig. 1:**
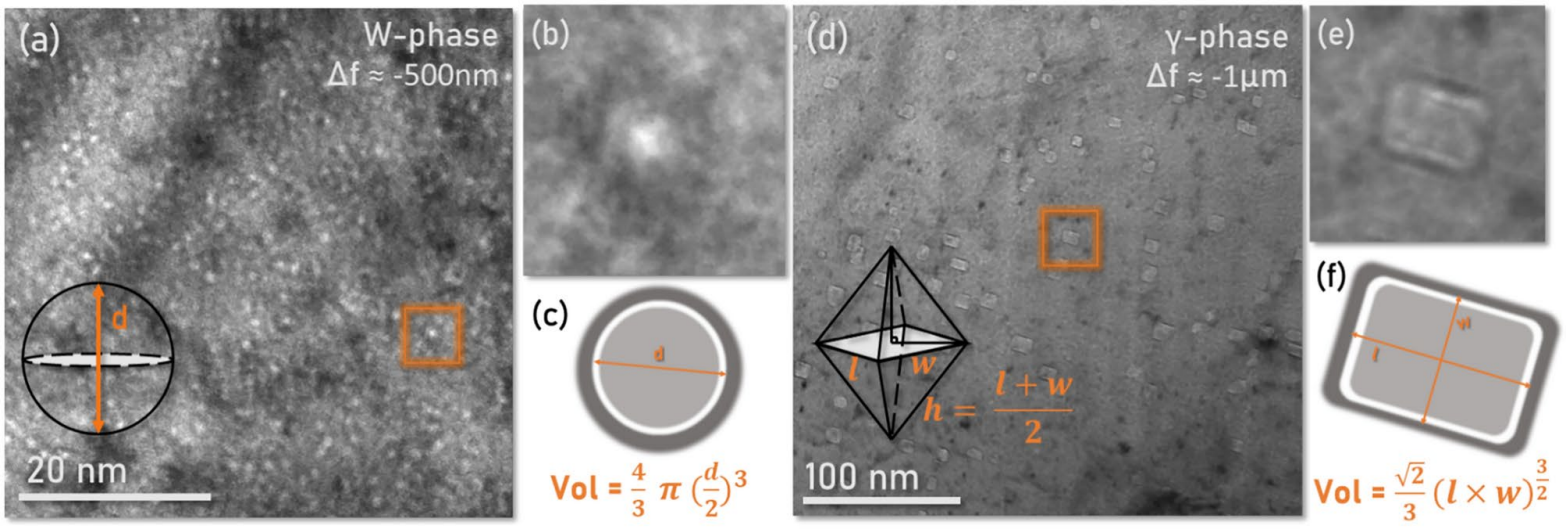
Fresnel fringe contrast from spherical and prismatic cavities in the TEM under-focus condition. Measurements of cavity size are generally taken as the inside of the first ‘dark’ ring. (**a**) example of small spherical cavities in a tungsten grain with a higher magnification view of a single cavity in (**b**).(**c**) Highlights the method for measurement of spherical cavity diameter and thereby volume. (**d**) Example of larger faceted prismatic cavities in a γ-phase grain with a higher magnification view of a single faceted cavity in (**e**). (**f**) Highlights the method for the measurement of cavity size and thereby volume.

Quantification of the defects present in irradiated specimens requires methodical characterization as well as an understanding of the underlying physics at play. Traditionally cavities are imaged in TEM mode by varying the defocus condition to either ‘over-focus’ or ‘under-focus’ conditions to better visualize the cavity. Rather than truly visualizing the edges in the cavity, the microscopist is instead viewing a series of sinusoidal phase interference near the matrix/cavity interface which results from a defocused beam channeling differently through the sample versus the void space of the cavity. These features are commonly called Fresnel fringes, and produce the characteristic contrast seen in Fig. 1 in the under-focus condition. These bright field TEM micrographs in the under-focus condition highlight the characteristic size and morphology of defects found in the bulk phases. The diameter of these cavities are then measured as the inner diameter of the first dark ring in this defocus condition, and require an objective defocus value of ≤ 750 nm to most accurately image cavities with a diameter of 2 nm or less^20^. No correction factor has been applied to the measurements of cavity size in this work.

Several collected works have focused on optimal series of imaging conditions depending on the characteristic size/shape of the defect as well as the associated TEM objective defocus values which should be employed for the most accurate defect measurement^20–22^, and more recent studies have pushed for direct imaging of cavities on strong zone-axis orientations in STEM mode as STEM produces a direct measure of void size^19^. Rather than assess current best-practice methods for the visualization of these defects, this work follows the TEM-based approach laid out in^20^.

### Bulk y-phase

**Fig. 2:**
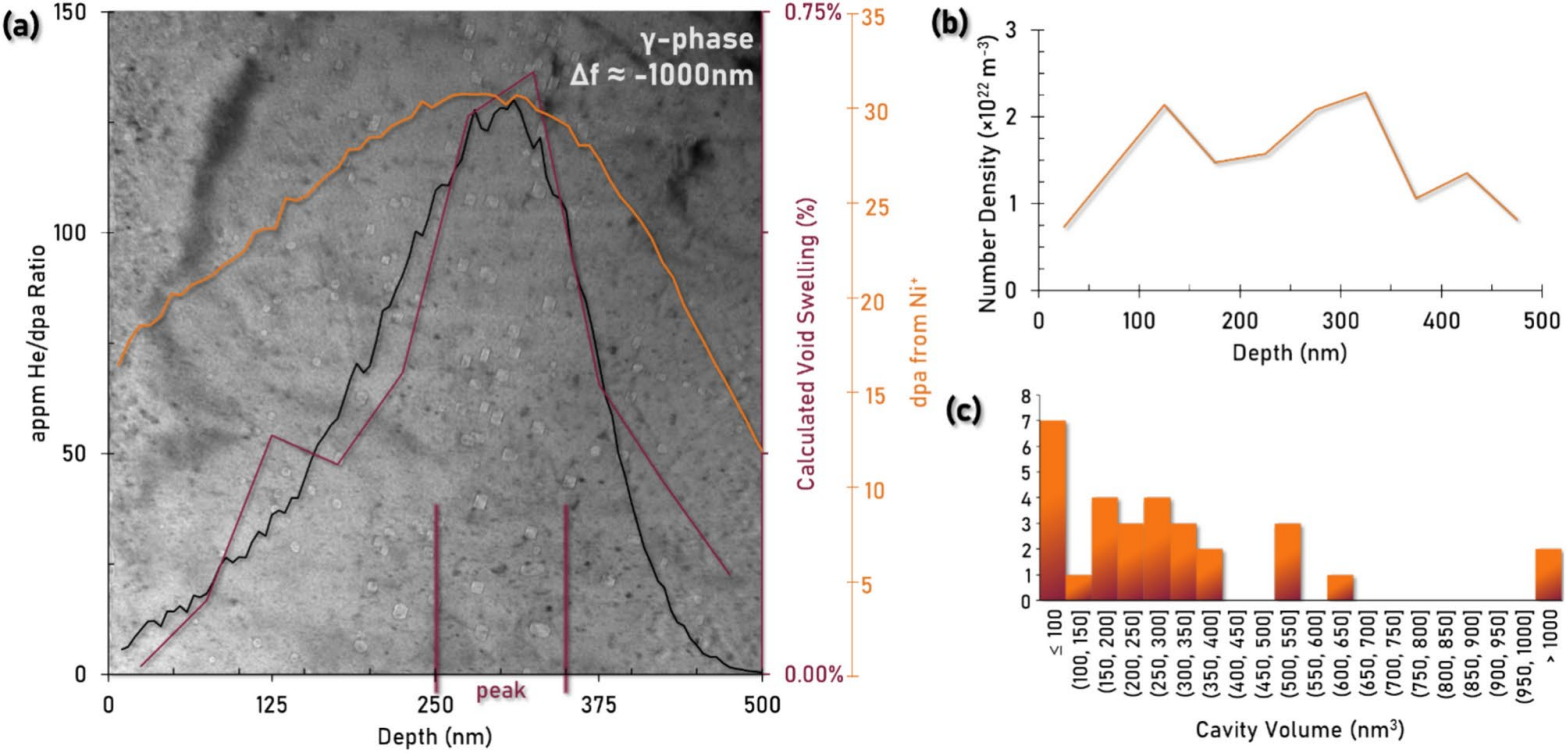
(**a**) under-focus TEM micrograph (Δf ≈ − 1 μm) of the top 500 nm of the γ-phase. Cavities can be observed throughout the depth of the implantation region with the highest apparent density of defects present at a depth of approximately 275–325 nm. The He/dpa ratio and dpa values have been plotted from SRIM simulation and the experimentally determined void swelling values have been overlaid on the micrograph to provide a numerical and spatial representation of the resultant γ-phase microstructure post-irradiation. (**b**) number density of cavities as a function of depth. (**c**) Histogram of individual cavity volumes at the peak swelling depth (275–325 nm).

For the calculation of defect density throughout the specimen depth in the γ-phase, a region of interest in the FIB foil was oriented to a kinematical condition, or away from any strong diffraction condition, and the bright field TEM micrograph in Fig. 2 was acquired with an objective defocus of approximately -1 μm. The corresponding raw data through-focal series can be found in the supplementary material (Fig. S1). This region was used to manually measure each He cavity and calculate individual cavity volumes. As the γ-phase presents two distinct cavity morphologies: spherical and faceted prisms, the volume of each cavity was calculated following the equations shown on Figs. 1c and f respectively. Curiously, there is an apparent transition in the morphology of the cavities from spherical to prismatic, then back to spherical as a function of depth below the irradiation surface.

Calculating the volume of a spherical geometry cavity is accomplished by measuring the diameter of the projected features, but capturing the shape and calculating the volume of a faceted cavity is less straightforward. In FCC materials, faceted voids are commonly found to adopt an octahedral shape^23,24^, and this work therefore approximates their volume as that of an irregular octahedron exhibiting different edge lengths ${\prime}l{\prime}, {\prime}w{\prime},$ and ${\prime}h{\prime}$. As the three side lengths needed to estimate the octahedral volume cannot all be determined from a single TEM projection, this volumetric calculation relies on two major assumptions: firstly, that the cavities shown in Fig. 2a are being viewed down their vertex direction projecting a rectangle with side lengths ${\prime}{l}{\prime}$ and ${\prime}w{\prime}$, and that the third axis, ${\prime}h{\prime}$, can be approximated as the average of ${\prime}{l}{\prime}$ and ${\prime}w{\prime}$. This is by nature an approximation rather than a true measurement and would necessitate a multi-projection analysis to deconvolute the exact shape of the voids within the γ-phase, but is thought to present a more accurate representation of both cavity morphology and volume as opposed to assuming an equivalent spherical volume.

To determine the changes in cavity density, and thereby estimated void swelling as a result of implantation depth, the γ-phase region of interest was broken down into 10 different 50 nm thick sub-sections for depth-tracking. The total number of cavities and the summed volume of all cavities in this sub-section were calculated and compared against the volume of the TEM foil in that sub-section. To calculate the volume of each sub-section, EELS thickness mapping was employed, and an effective atomic number of ~ 33.4 was used for thickness determination in the γ-phase. This value is an average based on the approximate atomic composition of the γ-phase found in prior EDS mapping (~ 60Ni, 30Fe, 10W at.%). Foil thickness was computed using Gatan’s DigitalMicrograph and measured in 6 points across each sub-section (top left, top center, top right, bottom left, bottom center, bottom right) then averaged to generate an effective foil thickness. The volume of the section is then computed from the average thickness in addition to the x-, and y-size of the sub-section.

The volume of each sub-section can then be used to produce a ‘calculated void swelling’ value (%) as well as to derive the number density (in m^-3^) of cavities as a function of depth. Statistics for these measured values as well as the average cavity volume as a function of depth have been provided in Table 1. The calculated swelling for γ-phase is found to increase to a peak value of approximately 0.68% at ~ 325 nm depth, then drops off quickly. The associated number density of defects at this depth is 2.3 × 10^22^ m^-3^, with an average cavity volume of ~ 300 nm^3^ (*n* = 30 cavities). To aid in visualization, the void swelling has been plotted as a function of depth in each sub-section in red in Fig. 2a*,* and plots of the number density of cavities and the average cavity volume have been provided in Figs. 2b and c respectively. Notably the low number density of cavities within the γ-phase presents too few cavities in each sub-section to gain a statistically significant view of cavity size dispersion at each depth for a reliable average size determination. The measured swelling values have been provided alongside the predicted dpa from the Ni^+^ ion irradiation as well as the He/dpa ratio from SRIM simulation for comparison in Fig. 2a. These values have been plotted to provide a depth-resolved representation of the incurred displacement damage (dpa) as well as the predicted profile of He^+^ implantation with respect to the Ni^+^ dpa and appm He/dpa ratio, due to their influence on void swelling in materials. Helium increases the binding energy of vacancies in a vacancy cluster, thus He increases the stability of the cluster and promotes cavity nucleation. The He/dpa ratio is a useful parameter in understanding and comparing He effect in composite materials where each phase experiences different He production rates, also in comparing He effect under different neutron spectra with different He production rates. The depth-dependent profile of the calculated void swelling curve is noted to follow the trend of dpa and He/dpa curves as expected.

**Table 1:** Characterization of cavity formation in the γ-phase as a function of depth below the irradiation surface.

Depth (nm)	# Cavities	# Density (× 10^22^ m^-3^)	Avg. Volume (nm^3^)	Swelling (%)
0–50	7	0.73	13.1	0.01%
50–100	16	1.4	58.1	0.08%
100–150	25	2.1	126.8	0.27%
150–200	18	1.5	161.3	0.24%
200–250	20	1.6	217.4	0.34%
250–300	27	2.1	304.0	0.63%
300–350	30	2.3	299.4	0.68%
350–400	14	1.1	310.4	0.33%
400–450	18	1.3	160.7	0.22%
450–500	11	0.82	138.0	0.11%

### Bulk W phase

**Fig. 3:**
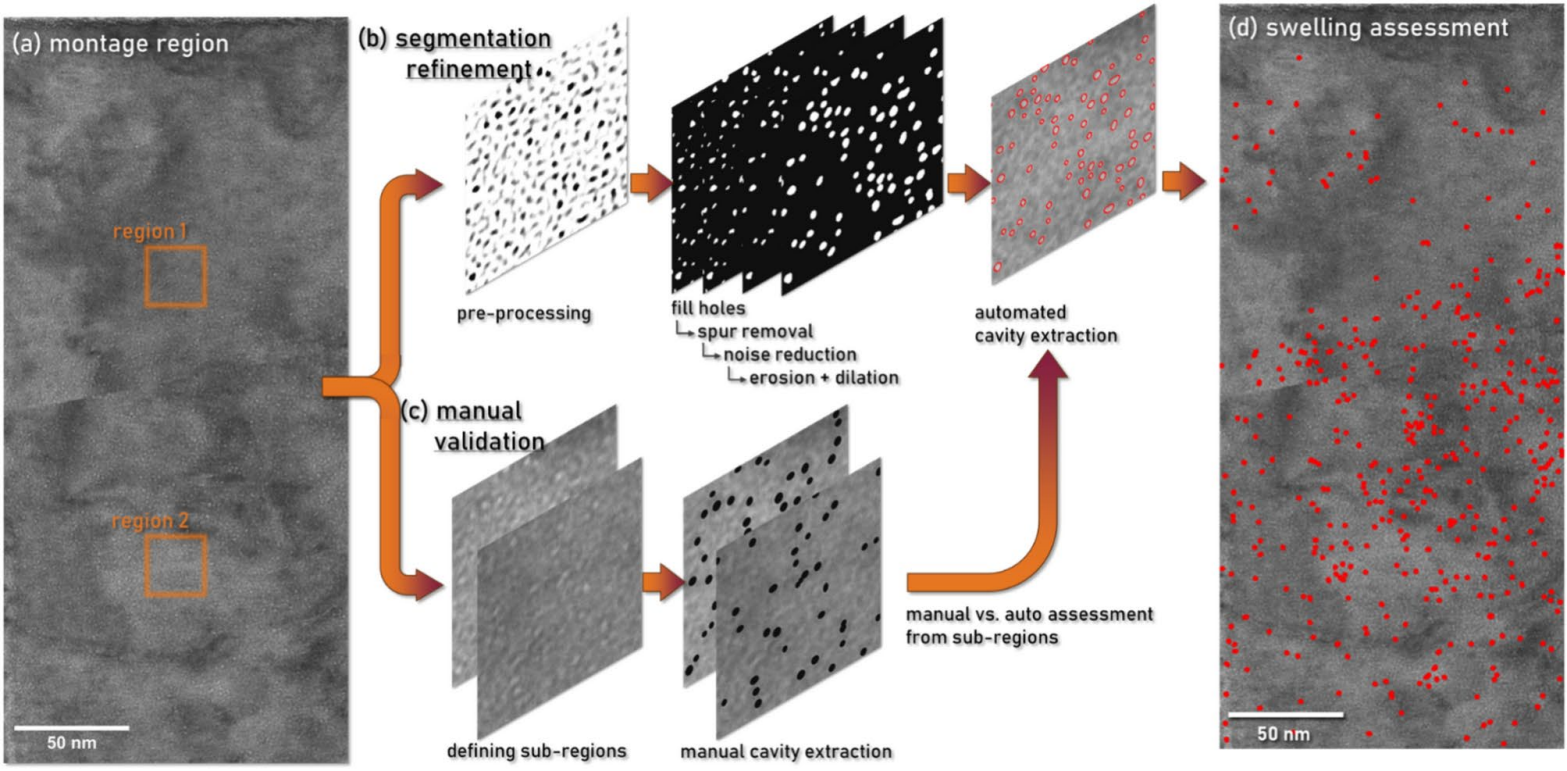
Flowchart of irradiated W cavity segmentation using the in-house developed image processing algorithm. (**a**) Montaged under-focus TEM data from W phase. (**b**) Refinement of the segmentation process for automated analysis detailing the pre-processing steps to correct non-uniform background intensities. (**c**) Manual extraction and measurement cavities from small sub-regions for benchmarking and validation. (**d**) Final swelling result for entire montaged region with cavities circled in red.

An identical analysis has also been performed for the W phase. As tungsten is a heavy element and the expected feature size is small, a suitably thin region (on the order of 50 nm thick or less) must be identified. Once this region was located, the grain of interest was oriented to a kinematical condition, and a series of high magnification under-focus micrographs were acquired from this W phase grain and stitched together into Fig. 3a. Due to the small size of the cavities in W, it was necessary to acquire multiple high magnification micrographs and montage them to resolve individual cavities over the implantation depth. Raw data from the TEM through focal series of this region has been provided in the supplementary material (Fig. S2). Figure 3a exhibits a high apparent density of cavities throughout the implantation depth. To avoid the extensive manual measurement of the high density of cavities across the region, an in-house image processing algorithm was developed to automatically process and segment cavities from acquired images. Image preprocessing was first applied to correct non-uniform background intensity and enhance cavity visibility. A pre-processing averaging filter was used to smooth the image and create a uniform background. This background was subtracted from the original image, resulting in a high-contrast gray-scale image where cavities stand out clearly against a bright background, as shown in Fig. 3b. The grayscale image was then converted to a binary format to effectively identify cavities. This conversion isolated the cavities as distinct objects against a unified background. During this process, several noise reduction and cavity refinement steps were applied, including filling any holes within the objects, removing extraneous spurs, and filtering to eliminate noise. The white blobs in the binarized result were then identified as cavities and the resultant image was used to extract individual cavity location, size, and shape for statistical analysis. To validate these results, a manual benchmarking step was added. Due to the size of the dataset, two sub-regions were defined, shown in the orange boxes in Fig. 3a. These regions were manually evaluated to extract cavity number density and size, Fig. 3c.

The manual and automated segmentation results were then compared against one another to evaluate the performance of the segmentation. The results of this comparison are summarized in Table 2 with histograms of the size distribution for both methods included in the supplementary information (Fig. S3). In Region 1, the automatic algorithm’s average cavity area (0.82 nm^2^) closely matches the manual measurement (0.81 nm^2^) with a minor error of 1.2%. The number of cavities detected by the algorithm (31) is slightly lower than the manual count (36), showing an error of 13.89%. In Region 2, the average cavity area detected by the algorithm (0.89 nm^2^) is comparable to the manual measurement (0.91 nm^2^), with an error of 2.20%. However, the algorithm identified more cavities (78) compared to the manual count (63), resulting in an error of 23.81%. Overall, the results indicate that both methods produce comparable results in terms of average cavity area and count, despite some discrepancies. This comparison underscores the feasibility of using the automatic algorithm for cavity segmentation, which significantly accelerated the processing time. The benchmarked automated segmentation was then applied across the montage region to capture the cavities, producing a binarized result which was used to assess cavities across the entire region of interest, Fig. 3d. Notably, a final filter was applied to the segmentation to discard cavities with a diameter $<1$ nm. This applied size threshold drastically affects the cavity counting statistics but has been set at 1 nm to reduce the inherent uncertainty in identification of cavities beyond the confidence threshold of TEM.

**Table 2:** Comparison of manual and automatic cavity measurement of cavities in irradiated W-phase used during training of automated analysis model.

Location	Method	Avg. Area (nm^2^)	Error (%)	# of Cavities	Error (%)
Region 1	Manual	0.81	**1.2**	36	**13.9**
	Auto	0.82		31	
Region 2	Manual	0.91	**2.2**	63	**23.8**
	Auto	0.89		78	

The segmented result was then sliced into 13 different 25 nm thick sub-sections across the 325 nm deep region of interest for depth tracking. The diameter, and thereby volume of each individual cavity was determined by calculating the area equivalent diameter ($d=\sqrt{\frac{4A}{\pi }}$) from the binarized and segmented image and was then summed into a total void volume within each subsection to estimate the void swelling. EELS thickness mapping of the W region was also performed, with identical 6-point thickness averaging for each sub-section as was performed for the γ-phase calculations. Since the W phase is nearly pure W, the effective atomic number for EELS thickness derivation was chosen as the Z-number of W (74).

**Fig. 4:**
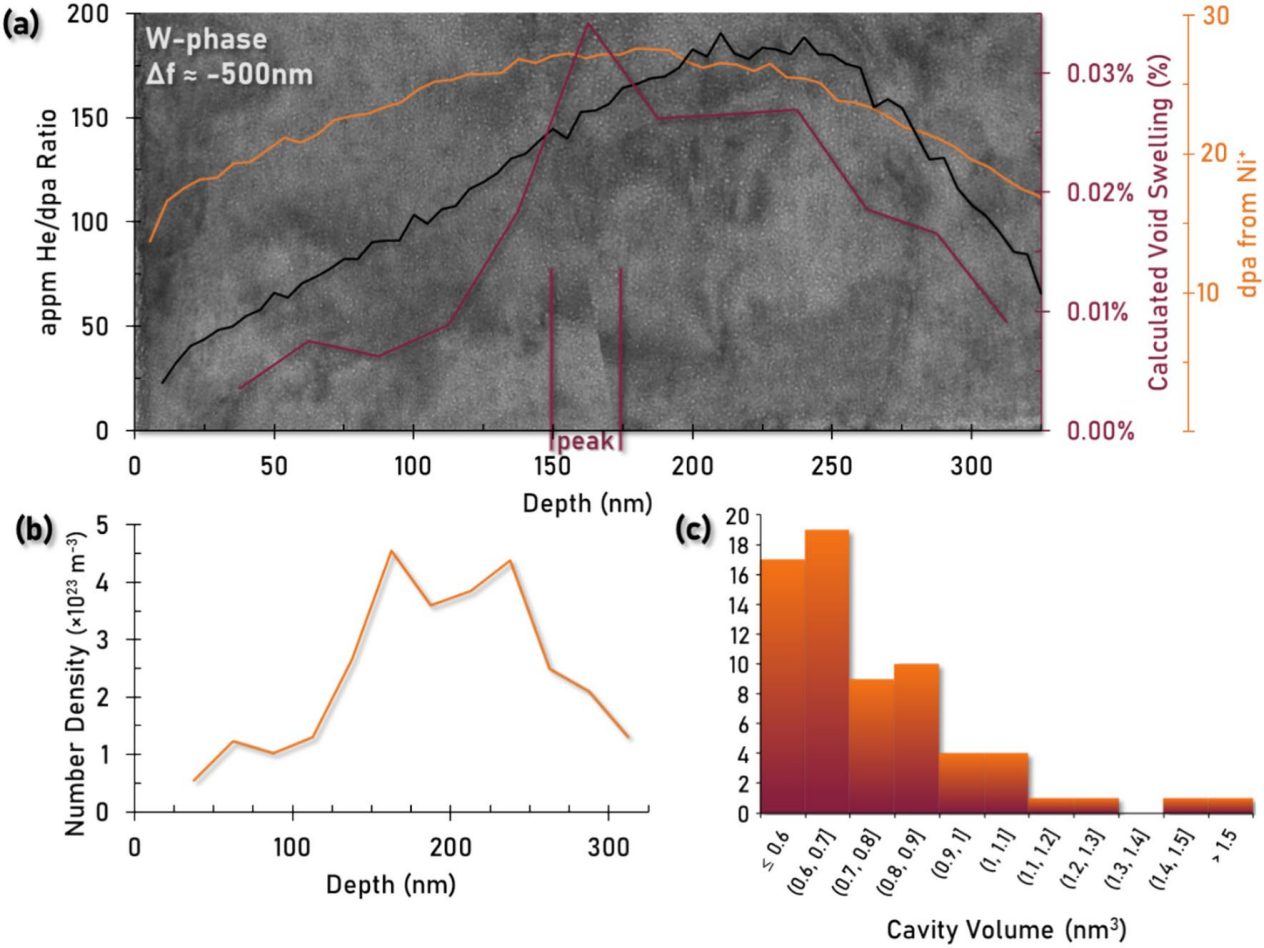
(**a**) under-focus TEM micrograph (Δf ≈ −0.5 μm) of the top 325 nm of the W-phase. Cavities can be observed throughout the depth of the implantation region with the highest apparent density of defects present at 150–175 nm. The He/dpa ratio and dpa values have been plotted from SRIM simulation and the experimentally determined void swelling values have been overlaid on the micrograph to provide a numerical and spatial representation of the resultant W-phase microstructure post-irradiation. (**b**) Number density of cavities as a function of depth. (**c**) Histogram of individual cavity volumes at the peak swelling depth (150–175 nm).

The results of this calculated void swelling have been overlaid on Fig. 4a, in addition to the dpa from Ni^+^ irradiation and He/dpa ratio from SRIM simulations for comparison. Figures 4b and 4c show the depth dependence for the number density of cavities as well as the dispersion of cavity volumes at the peak swelling depth (150–175 nm). The peak swelling in W is calculated to be approximately 0.03% at a depth of 150–175 nm below the surface. At this depth, the number density of cavities is measured to be 4.5 × 10^23^ m^-3^ with an average spherical volume of approximately 0.75 nm^3^ (*n* = 67 cavities). Statistics for the depth dependence of cavity formation and density as well as the resulting calculated swelling have been provided in Table 3. From these calculations, W is noted to swell substantially less than that of the γ-phase. This result should be tempered with the existence of He cavities in W which are smaller than the resolution limit of the TEM.

**Table 3:** Characterization of cavity formation in the W-phase as a function of depth below the irradiation surface.

Depth (nm)	# Cavities	# Density (× 10^23^ m^-3^)	Avg. Volume (nm^3^)	Swelling (%)
0–25	-	-	-	-
25–50	8	0.55	0.64	0.00%
50–75	18	1.2	0.61	0.01%
75–100	15	1.0	0.61	0.01%
100–125	19	1.3	0.68	0.01%
125–150	39	2.7	0.69	0.02%
150–175	67	4.5	0.75	0.03%
175–200	53	3.6	0.73	0.03%
200–225	56	3.9	0.69	0.03%
225–250	64	4.4	0.73	0.03%
250–275	37	2.5	0.74	0.02%
275–300	31	2.1	0.79	0.02%
300–325	19	1.3	0.71	0.01%

### IPB

**Fig. 5:**
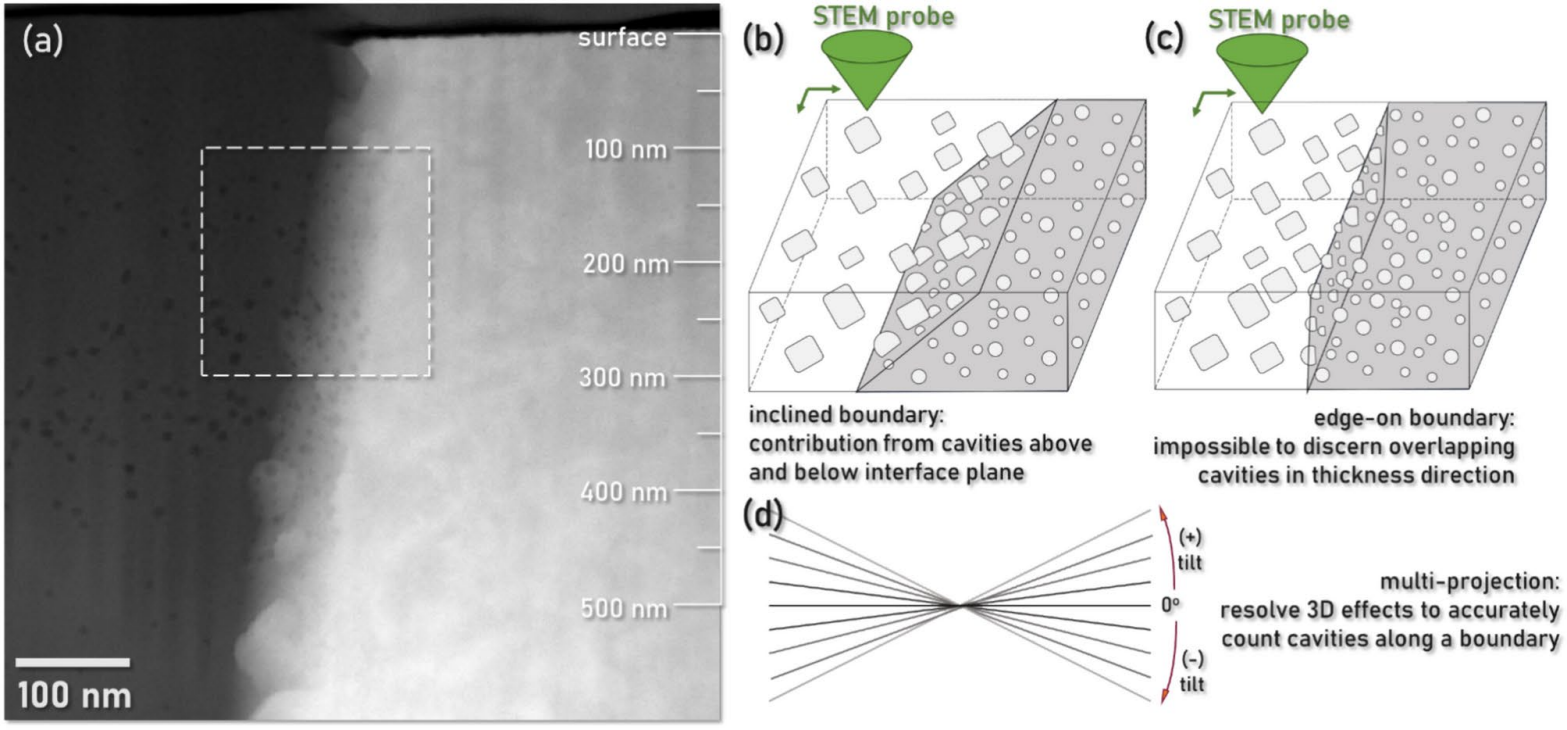
Analysis of cavities at an ion irradiated IPB. (**a**) HAADF STEM micrograph of the boundary region between the γ-phase (left) and the W-phase (right). A bounding box has been drawn to show the region of interest for detailed density calculation. (**b**) Schematic of the contribution from overlapping cavities at an inclined interface. (**c**) Schematic of overlapping cavities in the thickness dimension with a perfectly edge-on interface with respect to the electron beam. (**d**) Schematic of a multi-projection approach to deconvoluting projection effects for cavity density calculation.

Analysis of the cavity segregation at the interphase boundaries between the W and γ-phase is a more involved process than bulk grain cavity quantification. Rather than the derivation of a percent swelling in m^-3^, it is instead necessary to derive a 2D ‘areal coverage’ for defects lying on the boundary plane. The morphology of defects on the IPB can be seen in Fig. 5a, where a series of dome-shaped He cavities sit along the γ-phase side of the boundary. This boundary also presents several nanoscale precipitates consistent with the hexagonal W_2_C type tungsten carbide identified in previous work with this material system^9^. Rather than an under-focus TEM image, this micrograph has been acquired using STEM high angle annular dark field (HAADF) conditions. As the specimen is suitably thin (~ 50 nm) and the interfacial cavities are comparatively large with sufficient contrast for easy differentiation, it was elected to calculate interfacial cavity density in STEM mode. These conditions also aid in minimization of diffraction effects.

The interface in Fig. 5a is significantly inclined with respect to the incident electron beam. While this provides an excellent qualitative view of cavity segregation at the boundary, using this orientation of the boundary to calculate a bubble areal density will lead to over-estimation of cavity density as there are cavities in the bulk γ-phase and W-phase grains above and below which do not truly lie along the boundary. A schematic of this effect is shown in Fig. 5b. While it is possible to separate out the overlapping features of both bulk grains by orienting the boundary to the edge-on condition, Fig. 5c, this orientation leads to the inability to differentiate features in the thickness dimension of the TEM foil and thereby under-estimation of cavity density. This under-estimation effect is noted to be magnified in the case of high boundary defect densities and thick specimens. It is instead more accurate to view the interface of interest from multiple projection angles to deconvolute the effects of projection and quantify only the cavities which sit on the boundary plane, Fig. 5d.

**Fig. 6:**
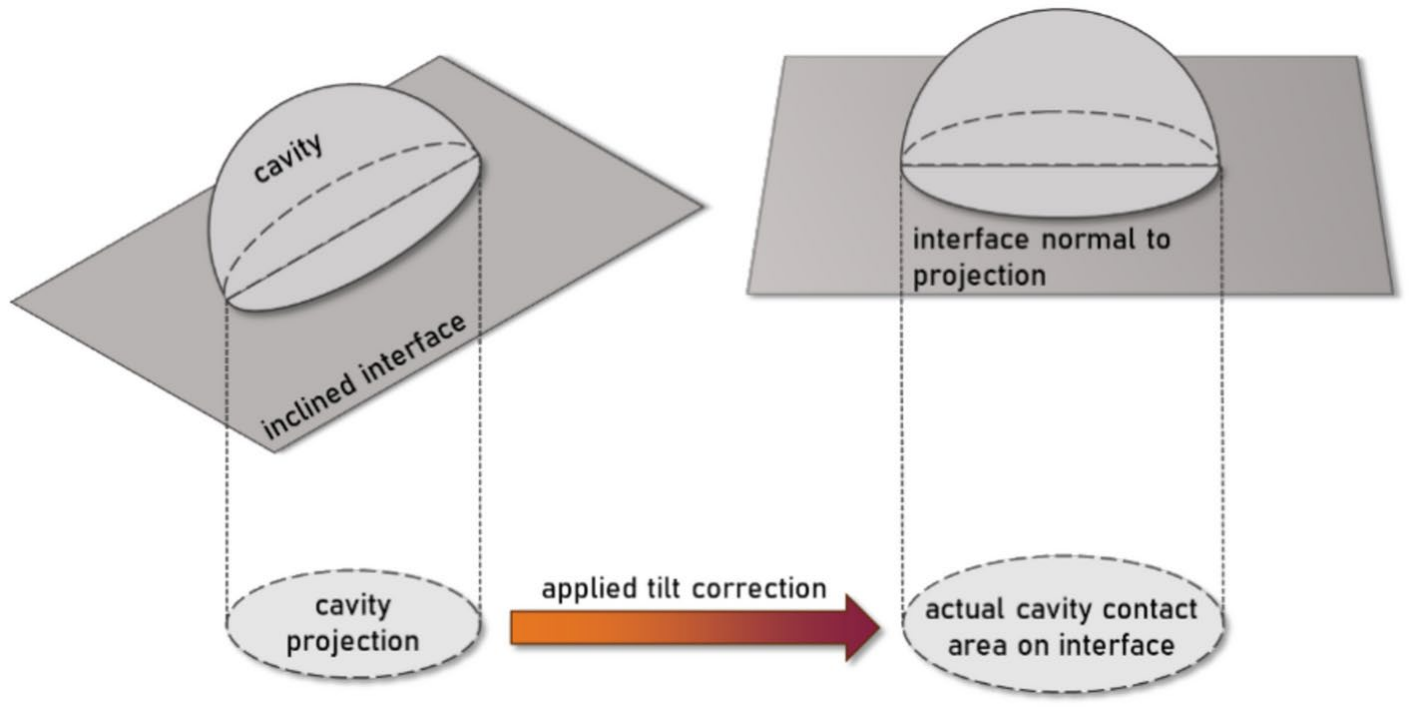
Schematic for the application of a tilt correction to more accurately calculate the contact area of dome-shaped cavities along a boundary plane in the case of an inclined projection.

This multi-projection analysis was performed for an IPB region of interest ranging from 100 to 300 nm in sub-surface depth, shown in the bounding box in Fig. 5a. The interface was imaged over an angular range of approximately 70° and the resulting images have been stacked together into a tilt series video provided in the supplementary material (*Video S1*). This region presents a IPB with limited carbide precipitation near the peak damage depth for both phases. The tilt series dataset yields *n* = 67 dome-shaped cavities lying along the boundary. Each cavity is assumed as a 2D ellipse for its contact along the boundary and has been fitted with a max and min Feret diameter for contact area determination. As all cavities are measured along an inclined boundary plane, a tilt correction has been applied to represent the true contact area of the cavities along the boundary as opposed to the reduced area noted from the projection of an inclined boundary. A schematic of this correction is shown in Fig. 6.

To determine the area of the boundary in this region of interest, identical EELS thickness mapping was performed on the W side of the boundary near the interface. The thickness of the W phase across this region was used in conjunction with the measured boundary overlap at the top and bottom of the region to calculate the area of the boundary. The boundary is assumed to be trapezoidal in shape, and yields a calculated interfacial area of approximately 1.7 × 10^4^ nm^2^. This value then allows the determination of areal number density as well as ‘areal coverage’. This areal coverage value is a representation of the corrected contact area of the elliptical dome cavity along the IPB plane and is a helpful analog in understanding defect coverage along a plane. This value is analogous to swelling value only in two dimensions. The calculated number density for cavities along the IPB plane is 4.0 × 10^15^ m^-2^ (*n* = 67), with an area coverage value of approximately 11.8%. The average contact area for the cavities along the boundary is found to be 29.7 nm^2^. It should be expressed that the total area probed with this technique is extremely limited, and therefore represents only a nanoscale section of 1 × IPB.

## Discussion

**Fig. 7:**
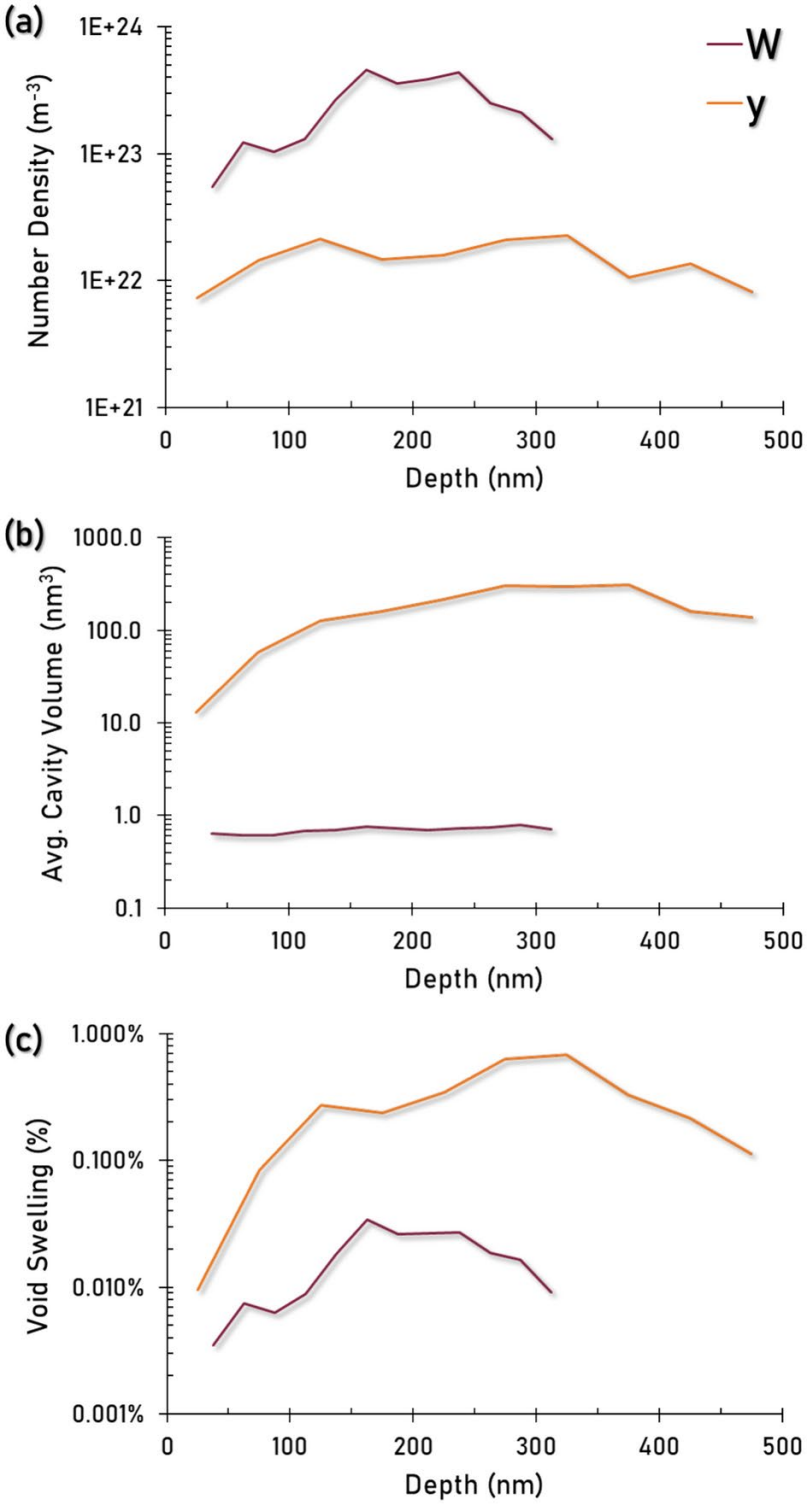
comparison of damage in the W and γ-phases in a WHA under identical irradiation conditions. (**a**) cavity density in both phases as a function of depth below the irradiation surface. (**b**) average cavity volume as a function of depth below the irradiation surface. (**c**) Void swelling in each phase as a function of depth.

To observe the characteristic irradiation damage microstructures in a 700 °C heavy ion irradiated tungsten heavy alloy, the W-phase, γ-phase, and IPB were quantitatively examined via S/TEM analysis. Figure 7 has been provided to graphically show the depth dependency on defect number density, average volume, and void swelling to represent the incurred damage in both phases, with Table 4 displaying the statistics of the calculated cavities at the peak swelling conditions. The W-phase presents a high density (10^23^ m^-3^) of cavities with an average spherical volume of approximately 0.75 nm^3^. It is noted that the volume of cavities in W remains relatively consistent throughout the implantation depth. Their characteristic 1–2 nm diameter and spherical morphology are in agreement with existing literature from similar temperature ion irradiations of tungsten^18,25^, and are notably an order of magnitude smaller than that of neutron irradiations done at 800 °C by Dürrschnabel et al.^26^. With the higher dose rates in ion irradiation and comparatively low irradiation temperature for the W phase (0.24T_m_), a high density of small cavities were expected and difficult to quantitatively characterize due to their high apparent density and size scale near the observable resolution limit in TEM thereby raising concerns for an exact capture of the swelling behavior within the bulk W phase.

**Table 4:** Damage statistics for WHA specimen under 700 °C Ni^+^ and He^+^ sequential ion irradiations.

	Peak Swelling / Areal Coverage (%)	Depth Range (nm)	Number Density	Avg. Volume (nm^3^)
W phase	0.03%	150–175	4.5 × 10^23^ m^-3^	0.75
γ phase	0.68%	300–350	2.3 × 10^22^ m^-3^	299.4
IPB	11.8%	100–300	4.0 × 10^15^ m^-2^	-

Swelling values for the W and γ phases are % swelling in m^-3^, and the IPB results are given as a % areal coverage.

In contrast to the W-phase, the γ-phase presents large, faceted voids at comparatively low density (10^22^ m^-3^) and an average volume orders of magnitude larger (~ 300 nm^3^) in the peak damage region. This defect morphology and size are consistent with prior studies on ion irradiated WHAs^18,19^ as well as with lower temperature studies on irradiated pure Ni and Ni-alloys^27–30^. Of particular note in the γ-phase is the peak void swelling from the applied irradiation conditions (~ 31dpa, 700 °C, 4.1 × 10^–3^ dpa/sec) is quite low at ~ 0.68%. Existing literature of self-ion irradiated pure Ni suggests a greater extent of swelling, even at more moderate fluences^28,29^. These aforementioned works note a 1.8% swelling in pure Ni (~ 4dpa, 500 °C)^28^ and 2.4% in pure Ni (~ 13dpa, 550 °C, 7.0 × 10^–4^ dpa/sec)^29^. Literature also suggests that the addition of gaseous ion species such as He/H will serve to magnify the effects of void swelling over pure self-ion irradiation^27^. There is a large scatter amongst Ni swelling values across different studies, at least partially owing to large differences in dose rates; but nonetheless one of the most compelling factors in these studies is the propensity for introducing solid solution alloying elements to Ni in effectively suppressing void swelling^28,30^. The inclusion of elements such as Fe, Co, and Cr in these works have been shown to lead to drastically reduced void diameters under identical irradiation conditions, which is a positive result for the application of WHAs as fusion reactor materials. It is then likely that the Ni–Fe-W containing solid solution γ-phase in this alloy is more effective in the suppression of void swelling than pure Ni. These results should be tempered with the comparatively low temperature stability of the γ-phase as compared to pure W, indicating that while Ni–Fe-W is demonstrated to perform well under such extreme irradiation conditions, it still exhibits substantially higher defect mobility than its W-phase counterpart.

The relative differences between the cavity sizes and densities can be conceptualized by looking towards the crystal structures and irradiation temperature regimes for each respective phase. BCC crystal structures in general present lower degrees of void swelling than their FCC counterparts, but this factor becomes more complicated when considering dissimilar compositions, displacement rates, and thermal properties^31^. Of particular interest is that the BCC W-phase is experiencing the applied irradiation conditions at a comparatively low fraction of its respective melt temperature (~ 0.24 T_m_) while the FCC γ-phase is at approximately 0.56 T_m_. In general, as homologous temperature for irradiation increases so does the vacancy diffusion rate. This promotes the nucleation of cavities. As irradiation continues, the cavities grow. As temperature increases further, dissociation of monovacancies from cavities can occur, particularly from the smaller cavities since the binding energy of vacancies in a cavity decreases as the cavity size decreases, and these dissociated monovacancies can be captured by the larger cavities, leading to void growth. With this in mind, it is shown that in these dual phase materials, both phases are essentially experiencing two different temperature regimes for the same applied irradiation conditions. This shows W to be in the cavity nucleation regime, experiencing a classic low temperature irradiation response by nucleating a high density of 1–2 nm diameter cavities with a comparatively low thermal driving force for their coarsening. Alternatively, the γ-phase is in the void growth regime, characterized by the formation of a lower density of large voids at the expense of consuming small cavities. These morphological differences are worth noting as it exhibits the curious ability of multi-phase materials to express phase dependent microstructural evolution under the same applied irradiation conditions.

While quantifying the defect population in the W and γ-phase bulk grains are necessary in understanding behavior of the WHA system, it is essential to observe damage segregation at the bi-phase interface. Strong interfacial bonding of the W and γ-phases gives rise to the ductile phase toughening (DPT) effect in WHAs^10,16^, and loss of this high interfacial cohesion will ultimately reduce WHA effectiveness. While interfaces are often sinks for irradiation damage, the one-sided nature of the defect segregation along the IPB speaks to the comparative mobility of defects in the W and γ-phases. It is theorized that while the γ-phase serves as an excellent ductile phase toughening agent in the pre-irradiated condition, that it is calculated to swell substantially more than W under the same irradiation conditions, possess lower inherent thermal stability, and generate less favorable transmutation products^14^. The derivation of a ~ 11.8% area coverage of cavities along the IPB planes under the imposed irradiation conditions will reduce the effectiveness of the DPT effect, likely resulting in measurable ductility and fracture toughness reduction. Added to this concern of WHA embrittlement from IPB cavity segregation, prior analyses of this alloy have identified the formation of deleterious carbides at IPBs which selectively embrittle WHAs in the presence of trace carbon contamination^9^. While the γ-phase and W-phase may perform adequately in isolation with 0.68% and 0.03% swelling values respectively, the development of boundary-localized precipitation and the accumulation of 11.8% area cavity coverage selectively at key sites for WHA mechanical performance is concerning. 3D void swelling and areal coverage values are also, by nature, temperature dependent. At temperatures in excess of 700 °C, emission of monovacancies from the ductile-phase and IPB cavities may occur which lead to desorption and removal of cavities from the ductile phase and IPBs to a free surface. It is therefore necessary to conduct future research over a variety of irradiation temperatures to determine the nature of cavity morphology and mobility.

The imposed irradiation conditions do represent a significant damage threshold at 5 years of simulated service, but the characteristic nature of this damage points to behavioral phenomena which are concerning for the application of these alloy systems in the fusion environment. Additionally, while the mechanisms which contribute to degradation of mechanical properties have been elucidated, the overall magnitude of the degradation remains to be solved, and is currently under investigation via micromechanical testing targeting unirradiated and irradiated IPBs in this alloy. To alleviate the proposed embrittlement through engineering of the WHA, literature suggests cavity growth may be suppressed through the addition of solid solution alloying elements in Ni^28,30^ as well as the inclusion of nano-dispersed oxides as is done for ODS steels. While these are both valid routes for the improvement of a W–Ni-Fe alloy, it may also be prudent to employ alternative ductile phase compositions with a higher temperature stability and lower He production rate, even if a moderate degree of ductility must be sacrificed.

These displayed results indicate performance issues with the prolonged use of a Ni–Fe based ductile phase due to increased swelling and high mobility of defects to crucial interphase boundaries. While these results are concerning for the application of a W–Ni-Fe WHA, it is clear that at present no material system currently exists which demonstrates the ability to withstand the fusion environment indefinitely. This study has instead endeavored to provide mechanistic insight into the nature of characteristic irradiation damage features in a material proposed to withstand the fusion environment and has quantitatively observed the incurred damage to aid in extrapolation of material performance. This process for rigorous examination of candidate materials under the simulated reactor environment is essential for material selection and performance evaluation. Bulk-scale material assessment will always be necessary to advance understanding of performance *en masse,* but high-resolution material characterization of surrogate irradiations is found to yield both phenomenological data to understand material performance and quantitative data to inform the predictive and extrapolative capacity of computational efforts on material behavior.

## Conclusions

A tungsten heavy alloy has been subjected to the simulated fusion environment to quantitatively assess the incurred damage and aid in the prediction of material performance as a plasma facing material in a fusion reactor. This investigation has revealed that the Ni–Fe based ductile phase swells substantially more than that of pure W, and that its low melt temperature leads to high defect mobility and therefore a high degree of defect accumulation at the dual-phase interface at 700 °C. To numerically assess this interfacial segregation, an areal coverage value was derived, and it is determined that approximately 11.8% of the W to γ interface plane is populated with cavities. The peak void swelling of the W phase is 0.03% and of the γ phase is 0.68%. While the γ phase is noted to swell to a greater extent than pure W, this work also indicates the Ni–Fe-W containing γ phase swells less than pure Ni under similar conditions and also presents a lower overall activation. Cavity segregation to the IPB is significant and is expected to result in selective embrittlement of the material at the dual-phase interface, systematically lowering the effectiveness of the Ni–Fe ductile phase as a toughening agent. To understand the magnitude of this selective hardening under irradiation a micromechanical testing study of irradiated boundaries is currently underway. Future studies are also needed to determine the swelling behavior as a function of temperature.

## Materials and methods

### Materials/irradiation

The material of interest to this work is a 90W-7Ni-3Fe (wt.%) tungsten heavy alloy. This is a liquid phase sintered alloy consisting of hard single crystal BCC W spheres suspended in a matrix of ductile FCC Ni–Fe-W, referred to here as the γ-phase. Specimens of the WHA were prepared for irradiation via wire electrical discharge machining (EDM) and mechanically polished to remove the EDM layer and present a pristine surface for ion irradiation. Sequential ion irradiations with 1.2 MeV Ni^+^ self-ions and 90 keV He^+^ were performed at 700 °C at Texas A&M University. Due to the dual-phase nature of these alloys, the ion irradiation steps were tailored to produce a peak damage region at approximately the same depth in both the W and γ-phases, as well as presenting damage/He production values characteristic of 5 years of service in a fusion power plant. These irradiation conditions are identical to those employed in prior analysis of thermomechanically treated WHAs in^18,19^ and irradiation assisted precipitation studies in the same alloy in^9^. SRIM predicts these conditions to yield damage values of approximately 27 dpa and 0.48 at.% He in the W-phase; as well as 31 dpa and 0.39 at.% He in the γ-phase at depths between 168 and 295 nm below the surface^18^.

### STEM/EELS

Lamellae for STEM analysis were prepared for defect characterization analyses from the Ni^+^  + He^+^ exposed region. Regions of interest were capped in a Thermofisher Hydra G4 Helios dual-beam focused ion beam scanning electron microscope (FIB-SEM) with 500 nm of electron-beam W and 3 μm of Xe^+^ ion-beam W using a gas injection system and a metal–organic. The regions were then extracted using a standard liftout procedure. Due to the considerable difference in milling rate between the phases, a Ga-source FEI Helios 660 Nanolab dual-beam FIB-SEM was used for thinning as the Ga^+^ ion beam has a considerably finer profile than the Xe^+^ beam. The lamella was thinned at 30 keV ion energy to 100 nm using a high-to-low tilt thinning method. Interfaces were then targeted and thinned to electron transparency in both the W and W–Ni-Fe phase at the depth of interest (the surface to 500 nm below the surface) at 5 keV ion energy. Once electron transparency was achieved, a final polish was conducted at 2 keV ion energy to minimize damage from preparation.

A probe corrected JEOL GrandARM transmission electron microscope operated at 300 kV was employed to characterize the effects of the applied ion irradiations. Imaging of the specimen foils was conducted in both scanning (STEM) and conventional (TEM) mode. For visualization of defect cavities in the bulk grains, bright field TEM through focal series have been acquired at over-, under-, and in-focus conditions. To aid in quantification of the defect densities within the thickness of the TEM foil, regions of interest have been imaged over multiple orientations to generate a large-angle tilt series. These tilt series provide a three-dimensional visualization of the damage features present within the specimen, which is found to provide superior feature identification and quantification, especially at interfaces such as grain boundaries and phase boundaries. Relevant details for the acquisition of these tilt series datasets can be found in ^32–34^. STEM HAADF has proven to be the more advantageous than TEM in tilt series collection and analysis for the interphase boundary region as it reduces the relative intensity of diffraction effects which occlude the cavities. These conditions magnify the effects of mass-thickness contrast by collecting highly scattered electrons. To generate volumetric defect density values from these tilt series datasets, electron energy loss spectroscopy (EELS) thickness mapping has been applied.

## Supplementary Information

Supplementary Information 1.

Supplementary Information 2.

## Data Availability

Data sets generated during the current study are available from the corresponding author on reasonable request.
