# Supplementary material for: Quantitative assessment of Ni+ and He+ ion irradiation damage in a tungsten heavy alloy under the simulated nuclear fusion environment
Source: Sci Rep. 2025 Feb 27;15:7066. doi: 10.1038/s41598-025-89532-w (PMC11868479; doi:10.1038/s41598-025-89532-w)
Supplement: Supplementary file 1 — Supplementary Information 1. [file 41598_2025_89532_MOESM1_ESM.docx]

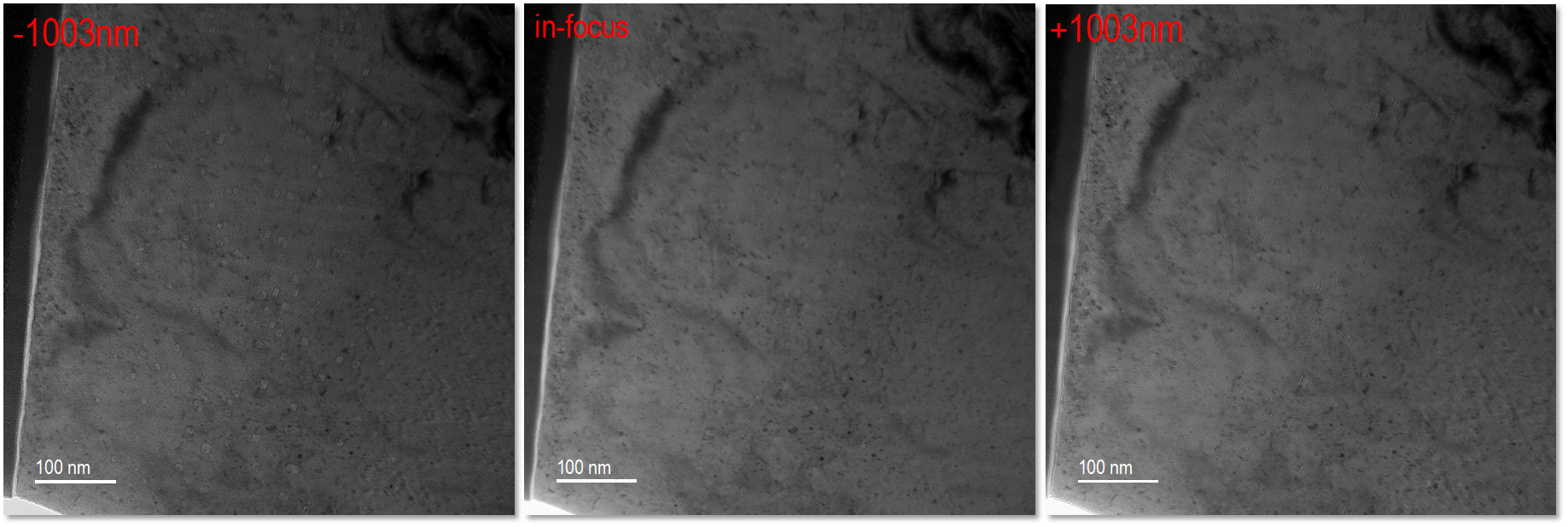


Figure S1: raw data through focal series of γ-phase grain.


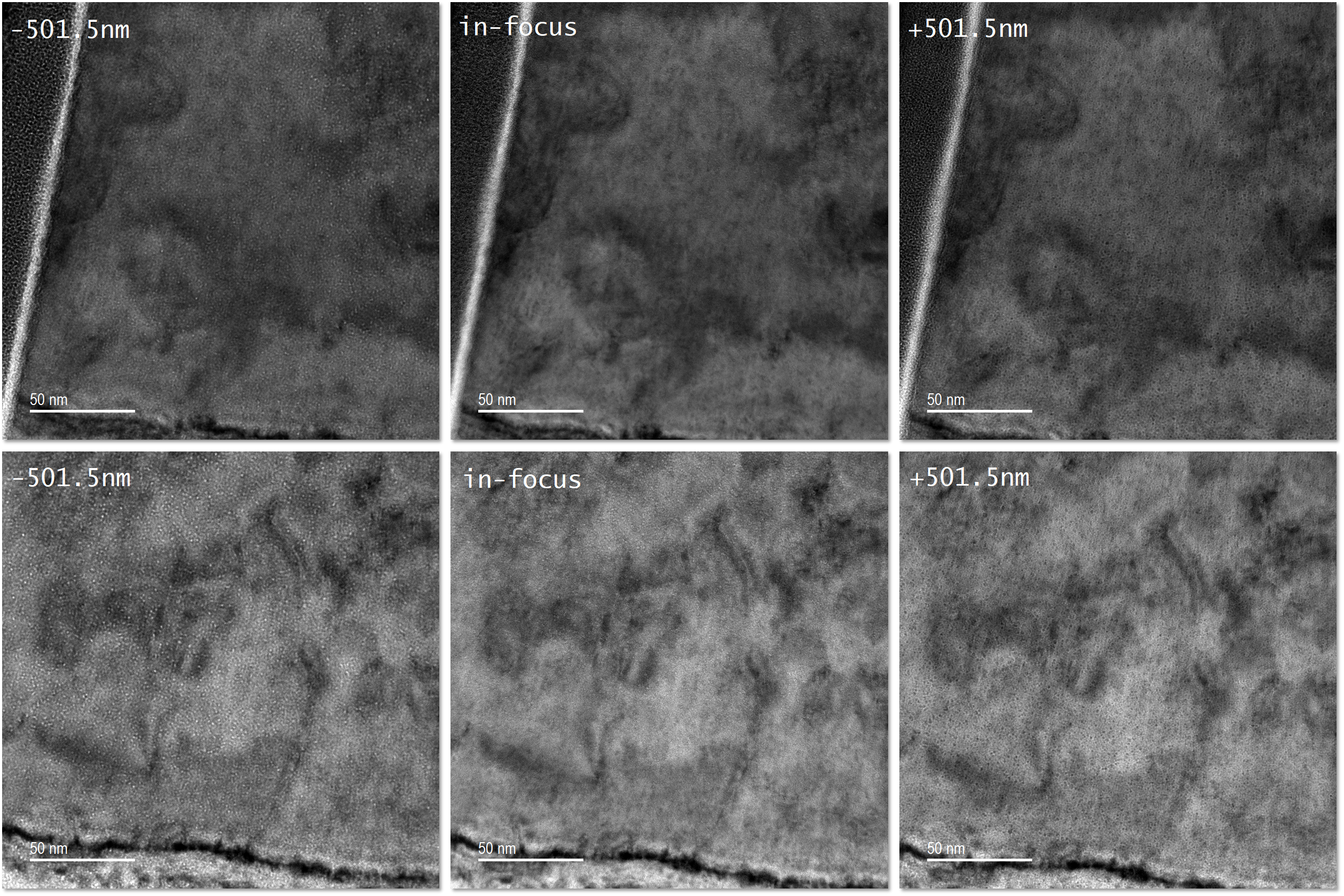


Figure S2: raw data through focal series of W-phase grain. Due to the high magnification necessary to image the nanometer-sized cavities in W, multiple images were taken and stitched together using DigitalMicrograph’s montage function to observe the depth dependence of defects.


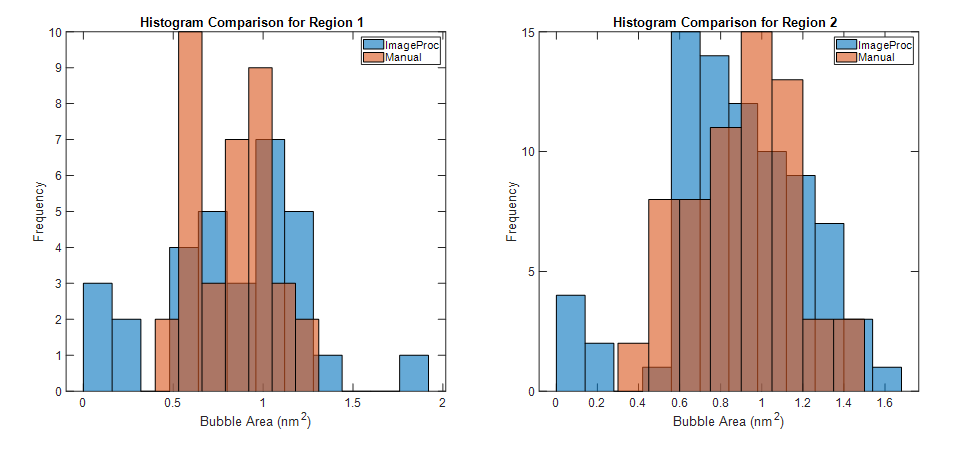


Figure S3: comparison of manual (orange) and automated (blue) cavity area distributions in the benchmarking regions.
