# Supplementary figures and images for: Quantitative assessment of Ni+ and He+ ion irradiation damage in a tungsten heavy alloy under the simulated nuclear fusion environment
Source: Sci Rep. 2025 Feb 27;15:7066. doi: 10.1038/s41598-025-89532-w (PMC11868479; doi:10.1038/s41598-025-89532-w)

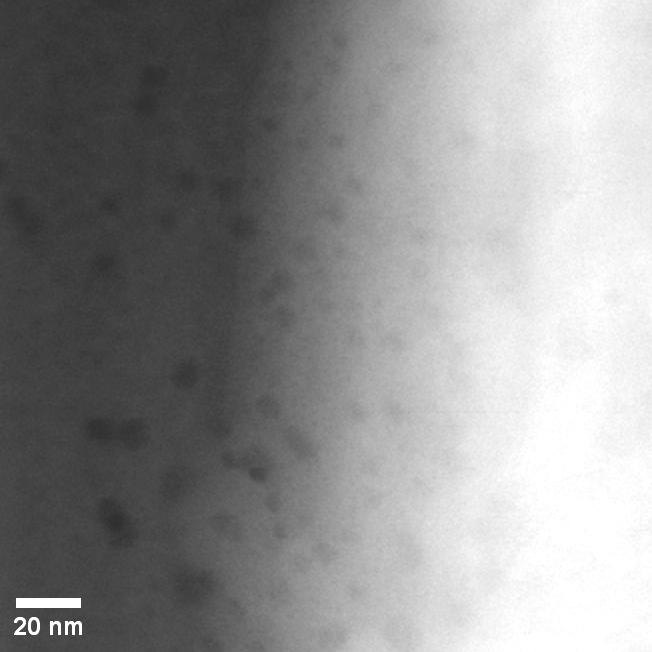

Supplement: Supplementary file 2 — Supplementary Information 2. [file 41598_2025_89532_MOESM2_ESM.gif]
